# Incidence and epidemiological patterns of meningitis among children and adolescents in Qatar: A primary care–based study

**DOI:** 10.5339/qmj.2026.59

**Published:** 2026-09-01

**Authors:** Hissa Mohamed Ghaith Al-Kuwari, Aisha Thani Al-Kuwari, Ahmad Haj Bakri, Mohamed Ghaith Al-Kuwari

**Affiliations:** 1 College of Medicine, Qatar University, Doha, Qatar; 2 Primary Health Care Corporation (PHCC), Doha, Qatar

**Keywords:** Meningitis, children, adolescents, epidemiology, primary health care, Qatar

## Abstract

**Background:**

Meningitis remains an important public health problem, particularly among children. In Qatar, despite recent improvements in surveillance systems, comprehensive national data on meningitis epidemiology among children and adolescents are still limited.

**Methods:**

A retrospective analysis was conducted using surveillance-confirmed meningitis data from the Primary Health Care Corporation (PHCC) electronic medical records and the Ministry of Public Health, Qatar surveillance system between January 2021 and December 2024. All confirmed meningitis cases among children and adolescents aged 0–19 years were included. Incidence rates were calculated by age group, year, and PHCC operational region using registered population denominators. Mean annual incidence rates were compared across regions using one-way analysis of variance.

**Results:**

A total of 228 meningitis cases were reported during the study period. Viral meningitis was the most common etiology (84.2%), while bacterial meningitis was less frequent, with Streptococcus pneumoniae (S. pneumoniae) being the main bacterial pathogen (11.0%). The overall incidence among children and adolescents was 9.31 per 100,000 population, with the highest burden observed among infants aged 0–1 year (50.44 per 100,000). Incidence decreased with increasing age. Regional variation was observed, with the Western region reporting the highest mean annual incidence (13.62 per 100,000), followed by the Northern and Central regions (*p* = 0.018). This variation was largely driven by viral meningitis.

**Conclusion:**

This study provides the first national overview of meningitis epidemiology among children and adolescents in Qatar. Viral meningitis predominated, with the highest burden among infants, while S. pneumoniae remained the leading bacterial cause. The findings highlight the need to strengthen surveillance, improve data integration, and sustain vaccination efforts in line with the World Health Organization (WHO) Defeating Meningitis by 2030 roadmap.

## 1. INTRODUCTION

Meningitis is an acute inflammatory disease of the meninges caused by a wide spectrum of pathogens, including bacterial, viral, mycobacterial, parasitic, and fungal agents.^1^ Among these, community-acquired bacterial meningitis represents the most clinically severe form, with *Streptococcus pneumoniae* (*S. pneumoniae*), *Neisseria meningitidis* (*N. meningitidis*), and *Haemophilus influenzae* (*H. influenzae*) type b accounting for the majority of cases worldwide. Additional pathogens predominate in specific geographic regions and high-risk populations, including neonates, pregnant women, transplant recipients, and older adults.^2^

Despite advances in antimicrobial therapy and vaccination, bacterial meningitis continues to impose a substantial global health burden, with case fatality rates ranging from 10% to 15% and a high prevalence of long-term neurological sequelae among survivors.^3,4^ In contrast, viral meningitis is more common and generally self-limiting, predominantly affecting infants and young children; however, its true incidence is likely underestimated because of underdiagnosis and mild clinical presentation. Viral meningitis is most commonly caused by enteroviruses (75–90%), followed by herpes simplex virus (HSV) and varicella-zoster virus (VZV), and often shows seasonal peaks during summer months.^5,6^ Importantly, bacterial meningitis requires urgent hospitalization, as outcomes are strongly dependent on timely diagnosis and prompt initiation of appropriate antimicrobial therapy.^7^

Global immunization programmes have led to marked reductions in meningitis-related morbidity and mortality, particularly among children. Conjugate vaccines against *S. pneumoniae* and *H. influenzae* type b have substantially reduced disease incidence and deaths in children younger than 5 years, contributing to the targets outlined in the World Health Organization (WHO) Defeating Meningitis by 2030 roadmap, which aims to eliminate epidemics, reduce deaths by 70%, and improve long-term outcomes among survivors.^8,9^ Nevertheless, sustained progress relies on robust disease surveillance and laboratory confirmation, the effectiveness of which varies across regions, including the Middle East.^10,11^

In Qatar, communicable disease surveillance has been strengthened through the implementation of the Surveillance and Vaccine Electronic System (SAVES) in 2020, enabling real-time case reporting and improved data quality. Despite these advances, epidemiological data on meningitis—particularly bacterial meningitis among children and adolescents—remain limited.^12,13^ To address this gap, the present study describes the epidemiological trends of bacterial and viral meningitis among children and adolescents aged 0–19 years in Qatar from 2021 to 2024, with the aim of informing public health strategies, vaccination policies, and clinical management.

## 2. METHODS

A retrospective analysis was conducted to estimate the incidence of meningitis among the Primary Health Care Corporation (PHCC), Qatar–registered population during the study period from January 1, 2021, to December 31, 2024. Data on surveillance-confirmed meningitis cases were extracted from PHCC electronic medical records (EMRs) and cross-verified with the surveillance system of the Department of Health Protection and Communicable Disease Control at the Ministry of Public Health. All meningitis cases were identified through the national surveillance system, which captures cases diagnosed across healthcare settings in Qatar, including primary, secondary, and tertiary care facilities. Diagnosis of meningitis is primarily established in hospital settings and subsequently captured through the national surveillance system. Cases were classified annually and included viral meningitis and bacterial meningitis caused by S. pneumoniae, N. meningitidis, and H. influenzae type b. Demographic characteristics and vaccination histories were obtained from PHCC EMRs to ensure linkage with the registered population and to address incomplete surveillance records. However, vaccination data were incomplete and were not included in the final analysis.

The study population comprised all individuals registered with PHCC as of December 31 of each study year (2021–2024). The population at risk included children and adolescents aged 0–19 years, stratified into the following age groups: 0–1, 2–4, 5–9, 10–14, and 15–19 years. All surveillance-confirmed meningitis cases reported between January 1, 2021, and December 31, 2024, within this age range were included. Demographic information was linked to PHCC records to ensure accurate attribution to PHCC operational regions.

The analysis included all confirmed bacterial and viral meningitis cases identified during the study period, including infections caused by *S. pneumoniae, N. meningitidis, H. influenzae* type b, and other specified or non-specified bacterial pathogens, as well as viral meningitis. Case classification followed national Ministry of Public Health surveillance definitions. These definitions are based on clinical and laboratory confirmation, including cerebrospinal fluid analysis, culture, and PCR, where applicable. Etiologies with very small case numbers were reported descriptively and excluded from regional incidence comparisons to avoid unstable estimates, while remaining included in overall case totals. Virus-specific etiological data (e.g., enteroviruses, HSV, VZV) were not consistently available in the surveillance dataset

Incidence rates were calculated by age group, year, and PHCC region using registered population denominators and are reported per 100,000 population. Mean annual incidence was estimated as the average of yearly rates across 2021–2024. Age-specific rates used corresponding population denominators, while overall incidence for children and adolescents aged 0–19 years was derived using population-weighted aggregates. Regional differences were assessed using one-way analysis of variance (ANOVA). Statistical significance was defined as *p* < 0.05.

This study is part of a larger project on the epidemiology of vaccine-preventable diseases and was approved by the PHCC Institutional Review Board (IRB) (reference number: PHCC/DCR/2022/01/005).

## 3. RESULTS

Between 2021 and 2024, 228 meningitis cases were reported at the primary health care level in Qatar among children and adolescents (0–19 years old). The number of cases increased steadily from 46 in 2021 to 101 in 2024.

Viral meningitis was the most common cause (192 cases; 84.2%), showing a rise after 2022 (55 cases in 2023 and 96 in 2024). Bacterial meningitis subtypes were less frequent, with Meningitis-BSP (25; 11.0%) and Meningitis-BNM (2; 1.0%). Other identified bacterial pathogens included *N. meningitidis* (4 cases), *H. influenzae* (2 cases), “Other bacterial” meningitis (3 cases), as in Table 1. 

Qatari nationals accounted for the largest proportion of cases (78; 34.2%). When grouped by region of nationality, Middle East and North African (MENA) countries represented the highest share (128; 56.1%), followed by Asia (61; 26.8%), Africa excluding North Africa (12; 5.3%), and other nationalities (27; 11.8%) (Table 2).

The overall incidence of meningitis among children and adolescents aged 0–19 years was 9.31 per 100,000. The highest incidence was observed among infants aged 0–1 year (50.44 per 100,000), followed by children aged 5–9 years (13.76 per 100,000) and 2–4 years (7.82 per 100,000), with a decline in older age groups to 0.79 per 100,000 among those aged 15–19 years. Viral meningitis was the predominant cause, particularly among infants (47.08 per 100,000), while bacterial meningitis, mainly due to S. pneumoniae (1.02 per 100,000), occurred at low incidence levels, with other bacterial pathogens contributing minimally (Table 3).

Meningitis incidence varied across PHCC regions during the study period (Table 4). The Western region recorded the highest mean annual incidence (13.62 per 100,000; 95% CI: 10.52–17.28), followed by the Northern region (8.07 per 100,000; 95% CI: 6.02–10.58), while the Central region showed the lowest incidence (6.34 per 100,000; 95% CI: 4.78–8.31). The overall regional difference in incidence was statistically significant (*p* = 0.018).

This regional variation was mainly driven by viral meningitis. The Western region had the highest viral meningitis incidence (9.48 per 100,000; 95% CI: 7.06–12.32), compared with the Northern (6.45 per 100,000; 95% CI: 4.58–8.87) and Central regions (5.55 per 100,000; 95% CI: 3.92–7.66), with statistically significant differences across regions (*p* = 0.021).

Bacterial meningitis subtypes occur at low incidence levels across all regions. Although *S. pneumoniae* meningitis was reported in each region, the observed differences were not statistically significant (*p* = 0.29). Other bacterial causes, including non-meningococcal bacterial meningitis, *H. influenzae*, and *N. meningitidis*, were rare and showed no significant regional variation (*p* > 0.05).

## 4. DISCUSSION

Meningitis remains a major global health challenge, with an estimated 2.51 million new cases and 236,000 deaths annually. The age-standardized incidence rate is 35.4 per 100,000 population, with a 30-day case fatality rate of nearly 15% among children aged 3 months to 14 years.^14^ This study provides the first nationwide assessment of age-specific and geographic patterns of meningitis among children and adolescents in Qatar, addressing a critical regional evidence gap. 

Viral meningitis was the predominant etiology, followed by bacterial meningitis, with *S. pneumoniae* as the leading bacterial pathogen. This pattern aligns with observations from high-income settings with high vaccination coverage. This etiologic distribution is characteristic of high-income countries with high vaccination coverage, where bacterial meningitis now accounts for less than 18% of cases.^15^ In contrast, data from Iraq (2007–2023) initially showed bacterial meningitis predominance, shifting toward viral meningitis in later years. These differences likely reflect temporal and contextual factors, including post-conflict recovery and vaccine expansion. The predominance of *S. pneumoniae* in Qatar further suggests sustained indirect protection from Hib vaccination, consistent with global declines in *H. influenzae* type b meningitis; for example, the Netherlands and the United States observed 35–75% reductions in *H. influenzae* cases following the type b conjugate vaccine introduction, respectively.^16,17^

The current study observed a decline in meningitis incidence during the COVID-19 pandemic, followed by a marked increase in 2023–2024. This pattern mirrors reports from other countries; for example, surveillance data from England demonstrated that laboratory-confirmed viral meningitis cases declined substantially during 2020–2021 when COVID-19 control measures were in place and increased again in 2022–2023 after societal restrictions were lifted, returning to pre-pandemic.^18,19^ This trend is plausibly attributable to post–COVID-19 epidemiologic dynamics, whereby reduced circulation of common viral pathogens during pandemic restrictions led to decreased population immunity—particularly among children—resulting in a rebound in infections once public health measures were relaxed. These observations underscore the pandemic’s indirect effects on infectious disease epidemiology and highlight the need for adaptive and resilient surveillance systems.

Qatari nationals accounted for the highest proportion of reported meningitis cases, indicating a relatively higher observed burden within this group. This pattern is consistent with prior surveillance in Qatar showing that confirmed viral meningitis was more prevalent among Qatari subjects compared to other nationalities, potentially reflecting differences in population structure, exposure risk, or health-seeking behaviors between nationals and expatriate groups.^13^ Regionally, the Western area consistently recorded the highest incidence, particularly for viral meningitis and S. pneumoniae, mirroring patterns observed for other communicable diseases.^20^ Such geographic clustering may be influenced by population density, mobility, health awareness, healthcare access, or localized transmission dynamics and warrants further investigation using spatial and multilevel analytic approaches.

This study has some inherent limitations. The retrospective design was based on routinely collected surveillance and medical records, which may be incomplete or subject to misclassification. In addition, not all cases were laboratory confirmed and viral serotype data were not available. Virus-specific etiological data (e.g., enteroviruses, HSV, VZV) were not consistently available, limiting detailed characterization of viral meningitis. The findings should be interpreted in light of the PHCC-based population denominator, which may limit full generalizability to the national population. Nevertheless, the inclusion of approximately 1.9 million PHCC-registered individuals, representing around 60% of Qatar’s population, provides strong national coverage and internal validity for children and adolescents. In conclusion, this study provides the first national overview of meningitis epidemiology among children and adolescents in Qatar. Viral meningitis was the predominant cause, with the highest burden among infants, while *S. pneumoniae* remained the leading bacterial pathogen.

## 5. CONCLUSION

These findings support strengthening routine surveillance and ensuring real-time data integration between primary and hospital care, maintaining high vaccination coverage, and prioritizing targeted prevention efforts for high-risk groups, particularly infants. Such actions are essential to enhance early detection, guide resource allocation, and support national efforts aligned with the WHO Defeating Meningitis by 2030 roadmap.

## ACKNOWLEDGEMENTS

The authors would like to thank the Department of Communicable Disease Control at the Ministry of Public Health (MOPH) and the Business Health Intelligence Department at Primary Health Care Corporation (PHCC), Qatar, for their support in the conduct of this study.

## AUTHORS’ CONTRIBUTIONS

All authors contributed to the data analysis, reporting, and preparation of the manuscript.

## CONFLICT OF INTEREST

None of the researchers involved in this project have any conflict of interest to declare.

## DISCLOSURE OF AI USE

The author acknowledge that the Claude AI tool was used for language refinement.

## DATA AVAILABILITY

The data supporting the findings of this study are available anonymised upon reasonable request.

## FUNDING

No funding was received for this study.

## ETHICAL APPROVAL

This study forms part of a larger project on the epidemiology of vaccine-preventable diseases and was approved by the PHCC Institutional Review Board (IRB), reference number PHCC/DCR/2022/01/005.

## Figures and Tables

**Table 1.** Meningitis among children and adolescents aged 0–19 years by Aetiology at the PHCC level, Qatar, 2021–2024.

Aetiology	Year				
	2021	2022	2023	2024	Total
Meningitis – Viral	38	3	55	96	192
Meningitis – BNM (Bacterial, not meningococcus)	0	0	0	2	2
Meningitis – BSP (Bacterial, Streptococcus pneumoniae)	0	2	21	2	25
Meningitis – IH (Haemophilus influenzae)	1	0	0	1	2
Meningitis – Other bacteria	3	0	0	0	3
Neisseria meningitidis	4	0	0	0	4
Total	46	5	76	101	228

**Table 2.** Distribution of meningitis cases among children and adolescents aged 0–19 years by region of nationality at the PHCC level, Qatar, 2021–2024.

Region of nationality	Number of cases	%
MENA (incl. North Africa)	128	56.10%
Asia (South + East Asia)	61	26.80%
Africa (excl. North Africa)	12	5.30%
Others	27	11.80%
Total	228	100%

Percentages are calculated based on total reported cases.

**Table 3.** Average age-specific incidence of meningitis among children and adolescents aged 0–19 years at PHCC level, 2021–2024 (per 100,000 population).

Aetiology	0–1	2–4	5–9	10–14	15–19	Total (0–19)
Meningitis -Viral	47.08	6.78	11.53	2.55	0.13	7.84
Meningitis – BNM	0.56	0.00	0.00	0.00	0.13	0.08
Meningitis – BSP	0.56	0.52	2.06	1.09	0.53	1.02
Meningitis – IH	1.12	0.00	0.00	0.18	0.00	0.12
Meningitis – Other Bacteria	1.12	0.00	0.00	0.18	0.00	0.12
Neisseria meningitidis	0.56	0.52	0.17	0.00	0.00	0.02
Total	50.44	7.82	13.76	4.01	0.79	9.31

**Table 4.** Regional average mean annual incidence rates of meningitis among children and adolescents aged 0–19 years, Qatar, 2021–2024.

Aetiology	Central mean (95% CI)	Western mean (95% CI)	Northern mean (95% CI)	*p*-value
Meningitis – Viral	5.55 (3.92–7.66)	9.48 (7.06–12.32)	6.45 (4.58–8.87)	0.021
Meningitis – Bacterial streptococcus pneumoniae (BSP)	0.68 (0.28–1.41)	1.46 (0.83–2.34)	0.94 (0.49–1.63)	0.29
Meningitis – Bacterial non-meningococcal (BNM)	0.11 (0.00–0.63)	0.12 (0.00–0.69)	0.13 (0.00–0.71)	0.5
Meningitis – Haemophilus influenzae (IH)	0.11 (0.00–0.63)	0.00 (0.00–0.43)	0.13 (0.00–0.43)	0.68
Neisseria meningitidis	0.00 (0.00–0.43)	0.24 (0.06–0.62)	0.27 (0.07–0.69)	0.41
All meningitis (total)	6.34 (4.78–8.31)	13.62 (10.52–17.28)	8.07 (6.02–10.58)	0.018

Incidence rates are expressed per 100,000 population; CI: Confidence interval.
